# Targeting the Giant Cell Tumor Stromal Cell: Functional Characterization and a Novel Therapeutic Strategy

**DOI:** 10.1371/journal.pone.0069101

**Published:** 2013-07-26

**Authors:** Matthew R. Steensma, Wakenda K. Tyler, Allison G. Shaber, Steven R. Goldring, F. Patrick Ross, Bart O. Williams, John H. Healey, P. Edward Purdue

**Affiliations:** 1 Department of Surgery, Spectrum Health Medical Group/Michigan State University College of Human Medicine, Grand Rapids, Michigan, United States of America; 2 Center for Skeletal Disease and Tumor Metastasis, Van Andel Research Institute, Grand Rapids, Michigan, United States of America; 3 Department of Orthopaedics, School of Medicine and Dentistry, University of Rochester, Rochester, New York, United States of America; 4 Research Division, Hospital for Special Surgery, New York, New York, United States of America; 5 Orthopaedic Service, Department of Surgery, Memorial Sloan-Kettering Cancer Center, New York, New York, United States of America; University of Medicine and Dentistry of New Jersey, United States of America

## Abstract

Giant cell tumor of bone (GCTB) is a benign, locally destructive neoplasm, with tumors comprised of mesenchymal fibroblast-like stromal cells; monocytic, mononuclear cells of myeloid lineage; and the characteristic osteoclast-like, multinucleated giant cells. Hampering the study of the complex interaction of its constituent cell types is the propensity of longstanding, repeatedly passaged cell cultures to undergo phenotypic alteration and loss of osteoclast-inducing capacities. In this study, we employed a novel, single-step technique to purify freshly harvested stromal cells using a CD14-negative selection column. Using 9 freshly harvested GCTB specimens and the purified stromal cell component, we performed analyses for markers of osteoblast lineage and analyzed the capacity of the stromal cells to undergo osteoblastic differentiation and induce osteoclastogenesis in co-cultures with monocytic cells. Successful purification of the CD14-negative stromal cells was confirmed via flow cytometric analysis and immunocytochemistry. Osteogenic media upregulated the expression of osteocalcin, suggesting an osteoblastic lineage of the GCTB stromal cells. The effects of the Wnt pathway agonist, SB415286, and recombinant human bone morphogenetic protein (BMP)-2 on osteoblastogenesis varied among samples. Notably, osteogenic media and SB415286 reversed the receptor activator of NF-κB ligand (RANKL)/osteoprotegerin (OPG) expression ratio resulting in diminished osteoclastogenic capacity. Recombinant human BMP2 had the opposite effect, resulting in enhanced and sustained support of osteoclastogenesis. Targeting the giant cell tumor stromal cell may be an effective adjunct to existing anti-resorptive strategies.

## Introduction

Giant cell tumor of bone (GCTB) is a benign, locally aggressive neoplasm that arises within the epiphyseal regions of long bones, as well as axial sites such as the sacrum or spine [1,2]. Osteolytic on plain film radiographs, GCTB is capable of causing significant destruction of bone. The three main cellular components of the tumor resemble constituents of the normal bone microenvironment--namely, a mesenchymal fibroblast-like stromal cell; a monocytic, mononuclear cell of myeloid lineage; and the characteristic osteoclast-like, multinucleated giant cell [3–5]. Several features of stromal cells suggest their neoplastic role within GCTB. Most notably, they are highly proliferative, allowing *in vitro* propagation through numerous passages in monolayer cell culture [5–7], and they have demonstrated a capacity to form tumors when implanted in immune-compromised mice [8–10]. The presence of telomeric associations, chromosomal aberrations, varied ploidy states, and gene amplifications have all been described within GCTB stromal cells [11–15]; however, these cytogenetic abnormalities correlate poorly with the clinical grading systems and clinical course [16].

Although characteristically osteolytic, bone formation does occur in GCTB under certain circumstances. Scattered nodules develop within the neoplastic tissue in up to 30% of cases [17]. Secondary bone formation may also occur as peripheral reactive bone or through fracture healing, and more recent data have confirmed intra-tumoral bone formation as part of a reparative response to receptor activator of NF-κB ligand (RANKL)-targeted therapy [18,19]. In accordance with these observations, results from several studies suggest GCTB stromal cells are of osteoblast lineage. Data confirm that stromal cells produce mature bone nodules when implanted subcutaneously in immunodeficient mice, and that GCTB lung metastases can contain osteoid and mature lamellar bone [20,21]. Molecular profiling of GCTB stromal cells consistently demonstrates the expression of early osteoblast lineage markers, such as Runx2 and Osterix (Osx), as well as variable expression of type I collagen and alkaline phosphatase (ALP) [16,20,22–26]. However, osteocalcin, a marker of advanced osteoblastic differentiation, is notably absent in highly purified GCTB stromal cell populations, suggesting the presence of an intrinsic or extrinsic block to osteoblastic differentiation within the tumor *in vivo*, potentially related to factors produced by the stromal cell population and/or the osteoclast-like giant cells [20,26].

GCTB stromal cells play an essential role in the recruitment of the tumor-associated myeloid lineage cells and formation of the osteoclast-like giant cells [3,16]. Evidence supporting this role include the capacity of GCTB stromal cells to induce osteoclastogenesis *in vitro* in co-culture studies with osteoclast precursors [27], and the demonstration that the stromal cells produce a broad range of factors involved in recruitment and induction of osteoclast differentiation and activation, including RANKL, the master regulator of osteoclast differentiation [3,16,19,20,27–29].

To date, studies of GCTB stromal cells have employed cell populations purified through serial passaging of the tumor cells. The extended time in culture and repeated passaging, however, are associated with a progressive alteration in the original biologic activities and functional properties of the stromal cells, including a gradual loss in the ability of the stromal cells to induce osteoclasts when co-cultured with myeloid lineage osteoclast precursors [6,27]. In this study, we describe a novel, single-step selection technique that allows purification of freshly harvested stromal cells, as well as isolation of the CD14+ myeloid lineage cells from the excised tumor tissue. Using these isolated and purified cell populations, we tested the hypothesis that GCTB tumor stromal cells are of osteoblastic lineage, and we characterized the mechanism underlying their unique functional properties, including their ability to support osteoclastogenesis.

## Methods

### Tumor procurement

Nine GCTB specimens were freshly harvested in accordance with protocols and informed patient consent waivers approved by the Hospital for Special Surgery’s Institutional Review Board. Clinical information for each patient is shown in Table 1. The initial diagnosis was established via frozen section in the operating room and was later confirmed on permanent histologic examination. A board-certified pathologist reviewed each sample to confirm viability (>80% by nuclei counts on hematoxylin and eosin–stained sections) and tumor content (>90%) for each sample. Planned analyses were performed on each specimen as sample size allowed.

**Table 1:** Patient and tumor characteristics of harvested GCTB specimens.

**GCTB Sample #**	**Age (y)**	**Sex**	**Anatomic Location**	Size* (cm)	**Adjuvant Radiation**
1	40	M	Sacrum	8.2	Yes
2	42	F	Distal femur	6.0	No
3	39	F	Distal femur	7.0	No
4	28	M	Distal femur	5.7	No
5	24	F	Distal femur	6.0	No
6	51	F	Patella	2.0	No
7	36	M	Distal femur	6.0	No
8	18	F	Distal femur	4.5	No
9	43	F	Distal femur	5.0	No

* Maximal diameter determined based on preoperative radiographic measurement.

### Cell separation

**Figure 1:**
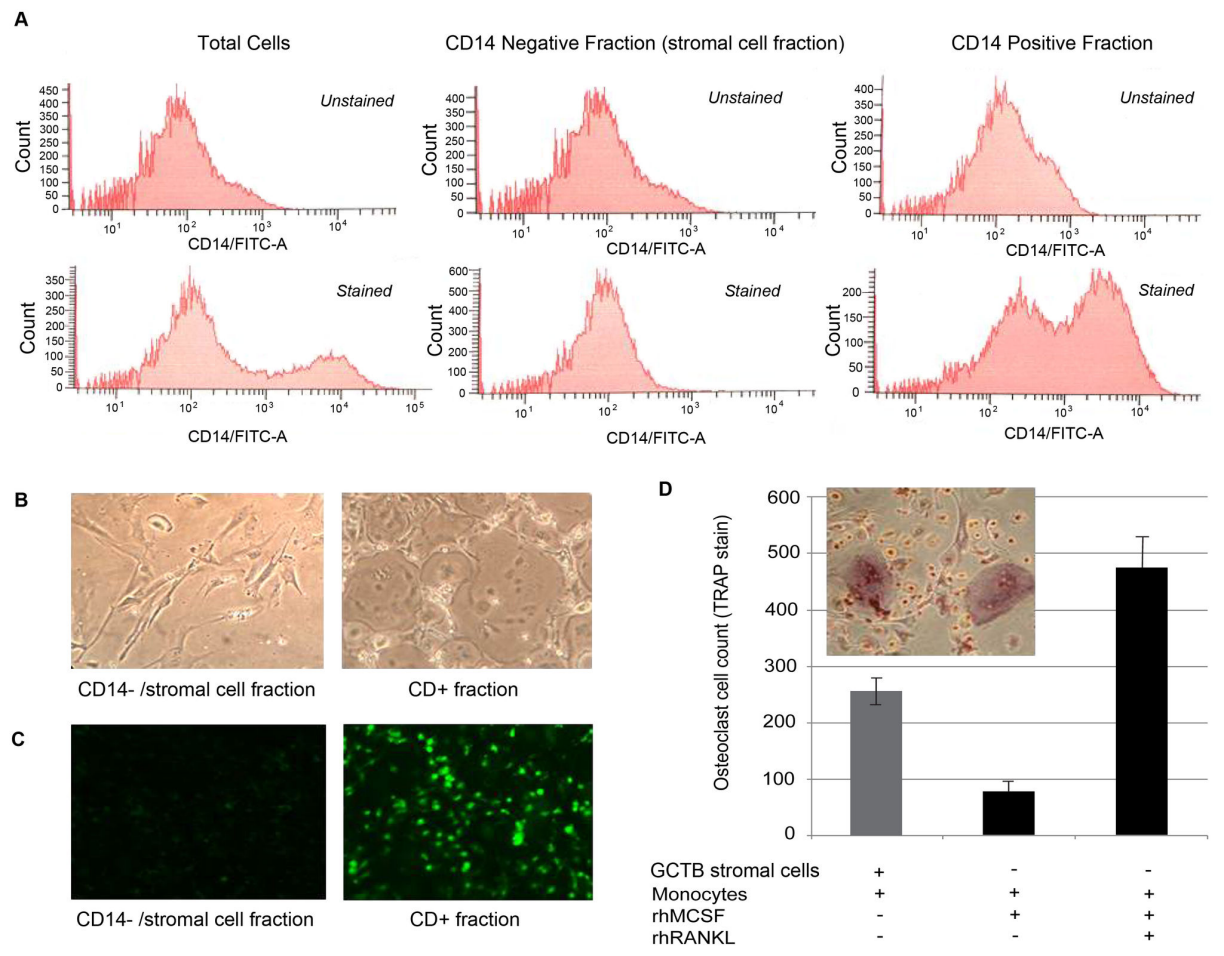
Separation of total GCTB cell digest into stromal and myeloid fractions. (A) Flow cytometry analysis of CD14+ cell population in total dispersed cells, stromal cell fraction and column retained fraction after CD14 negative selection column separation (top row: unstained control; bottom row: FITC-labeled anti-CD14 Ab). (B) Post-column separation phase contrast microscopy demonstrating morphology consistent with GCT stromal cells (left) and a combination of multinucleated giant cells plus monocytic, mononuclear cells (right). (C) CD14 immunofluorescence of cells grown on glass coverslips from the stromal cell (left) and column retained fractions (right). (D) Isolated GCT stromal cells maintain their ability to induce osteoclast formation in co-culture with human peripheral blood monocytes.

The freshly harvested GCTB tissue was minced and digested with 0.1 mg/mL type I collagenase (Worthington, Lakewood, NJ) in MEM-α for 1 hour with frequent agitation. Dispersed cells were passed through a 70 µM nylon filter and cells were counted. Stromal cells were isolated using a negative selection technique, in which total dispersed cells were labeled with magnetic beads bound to a monocyte marker, CD14 (Miltenyi Biotech, Auburn, CA), according to the manufacturer’s recommendations, then passed through a magnetized column. CD14 surface expression in the adherent and non-adherent fractions was characterized by analytical flow cytometry analysis (FACS Aria®) (anti-CD14 antibody ab28061; ABCAM®; Cambridge, MA) and immunocytochemistry according to manufacturer’s protocol. The initial non-adherent, CD14-negative fraction was highly enriched in GCTB stromal cells (Figure 1A, 1B, and 1C) and maintained the ability to induce osteoclastogenesis in a human monocyte co-culture system (Figure 1D; also described below).

### GCTB stromal cell differentiation

Freshly isolated GCTB stromal cells were plated in MEM-α supplemented with 10% fetal bovine serum (FBS) at 50,000 cells per well in a 12-well plate. Cells were grown in the presence or absence of the glycogen synthase kinase (GSK) 3β inhibitor, SB415286 (10 µM) (GlaxoSmithKline, Middlesex, UK) [30]; human recombinant bone morphogenetic protein (BMP)-2 (300 ng/mL) (Peprotech, Rocky Hill, NJ); or osteogenic media consisting of 10 μM ascorbic acid, 10^-8^ M dexamethasone, and 10 mM β-glycerophosphate in 10% FBS/MEM-α. Cells were grown under experimental conditions for 12 days with media changes occurring every 3 days. Cells were either harvested for RNA using the Trizol® (Invitrogen, Carlsbad, CA) extraction method, or incorporated into the osteoclastogenesis assay described below. Von Kossa staining was performed as described elsewhere [31].

### PCR and ELISA assays

Reverse transcription of purified RNA to cDNA was performed using a commercially available kit (Fermentas; Glen Burnie, MD). After performing quantitative real-time PCR (qPCR) using a Biorad ICycler platform and SYBR green master mix (BioRad, Hercules, CA), the relative expression levels for each gene were determined using the ΔΔCt method, normalized to the expression level of the housekeeping gene *GAPDH*. Intron-spanning primer sets were constructed for key osteoblastogenic and osteoclastogenic regulatory genes and markers (Table 2). Conditioned medium was collected at 2-day intervals, and levels of osteoprotegerin (OPG) protein were determined using a commercially available OPG ELISA kit (R&D Systems, Minneapolis, MN). Comparisons were analyzed statistically using the Mann–Whitney U test with post-hoc adjustment of the p value.

**Table 2:** Primers used in PCR assays to detect key osteoblastogenic and osteoclastogenic regulatory genes and markers.

**Gene Name**	**Forward Primer**	**Reverse Primer**
OPG	CCGCGTGTGCGAATGCAAGG	TGGGGTTCCAGCTTGCACCAC
RANKL	AAGGAGCTGTGCAAAAGGAATT	TGATGTGCTGTGATCCAACGA
Osteocalcin	CTGCGATGACACAGCAAATC	GGACTTTGCCTTCTTCCACA
Osterix	TTCTGACTGTCTGCCCAGTG	GCCTTGTACCAGGAGCCATA
ALP	CCACGTCTTCACATTTGGTG	GCAGTGAAGGGCTTCTTGTC
BMP2	ACATGGTTGTGGAGGGTTGT	CAACTGGGGTGGGGTTTT
BMP3	TAGAGTCTTGCGCTTGCAGA	GAAACAAAATGCATTGGCAGT
BMP4	TCCATGCTGTACCTGGATGA	GGAACGTGTGTGTGTGGTGT
BMP6	GCGCCAACTAAGCTAAATGC	TCCAAGGCAGAATGTGTGTC
BMP7	TGCCATCTCCGTCCTCTACT	GCAATGGAGGATCCAGAAAA
ALK2	CAAAATCCATCCGCAAGACT	GCTGGACAATGACAACAACG
ALK3	AGCCTCCAGACTCACAGCAT	CATGCCATGGGTAAAAACAG
ALK6	CCTGCGGGTTAAGAAAACAC	CTCTGCCCACAAACAGAAGA
BMPRII	TCTTTTCTTTGCCCTCCTGA	AGCAGGATGGTCCATGGTAG
Runx2	TCTGGCCTTCCACTCTCAGT	ATGAAATGCTTGGGAACTGC

### Western blotting

Freshly isolated GCTB stromal cells were plated at 5×10^6^ cells/well and exposed to vehicle or BMP2 or SB415286 for 0-4 hours. Protein extraction was carried out in 1X SDS lysis buffer (100 mM Tris pH 6.8, 2% SDS, 2.5% β-mercaptoethanol, 15% glycerol, 0.2 mg/mL bromophenol blue, 1 mM phenylmethanesulfonylfluoride (PMSF) (Sigma-Aldrich, St. Louis, MO) on ice. Samples were boiled for 5 minutes, loaded onto a 10% Tris-HCl precast gel (Biorad, Hercules, CA), and run at 200 V for 1 hour in 1X Tris/glycine/SDS buffer. Semi-dry transfer to a PVDF membrane (Millipore, Billerica, MA) was performed in 1X Towbin buffer, followed by blocking with 5% nonfat dry milk solution in phosphate-buffered saline (PBS)-Tween 20. Primary antibodies used were mouse anti-active-β-catenin, clone 8E7 (1:1000) (Millipore, Billerica, MA) or rabbit anti-Phospho-Smad1/5 (Ser463/465) (41D10) (1:1000) (Cell Signaling Technology, Beverly, MA); followed by secondary anti-mouse horseradish peroxidase (HRP) (1:10,000) (Sigma Aldrich, St. Louis, MO) or anti-rabbit HRP (1:10,000) (Sigma-Aldrich, St. Louis, MO). ECL detection reagent was applied (GE Healthcare Biosciences; Pittsburgh, PA) and digital images were acquired on a commercially available fluorescent imager (Biorad, Hercules, CA).

### Osteoclastogenesis assays

Freshly isolated, peripheral blood monocytes were obtained from human donors, as previously described [8], under protocols approved by the Hospital for Special Surgery’s Institutional Review Board. The monocytes were plated at a density of 6.25×10^5^ cells per well in a 12-well plate containing 5×10^6^ preplated GCTB stromal cells, and monocytes were also plated alone (6.25×10^5^cells/well) in the presence of 25 ng/mL macrophage colony stimulating factor (M-CSF) with or without 40 ng/mL RANKL (both Peprotech, Rocky Hill, NJ) as negative and positive controls, respectively. In all cultures, media and cytokines were changed every 3-4 days. After 10 days of culture, cells were stained for tartrate resistant acid phosphatase (TRAP) using a commercially available kit (Sigma-Aldrich) and triplicate counts of TRAP-positive multinucleated giant cells were performed. Comparisons were analyzed statistically using the Mann–Whitney U test with post-hoc adjustment of the p value.

## Results

### Isolation of GCTB stromal cells and characterization of osteoclast-inducing activity

Flow cytometry analysis of total dispersed tumor cells identified a population of CD14+ myeloid cells, and fractionation using anti-CD14 magnetic beads separated these CD14+ cells from the CD14-negative population with high efficiency (Figure 1A). The negatively selected CD14-negative cells displayed a uniform fibroblast-like, spindle cell morphology, whereas cells with monocytic or osteoclast-like, multinucleated giant cell morphology were visualized using light microscopy in the CD14+ fraction (Figure 1B). Confirmation of the efficiency and specificity of the column separation technique was also demonstrated using immunocytochemistry (Figure 1C). Importantly, the CD14-negative/stromal cell fraction exhibited consistent osteoclastogenic capacity as evidenced by induction of robust osteoclast formation when co-cultured with human peripheral blood monocytes (Figure 1D).

### Induction of GCTB osteoblast differentiation

**Figure 2:**
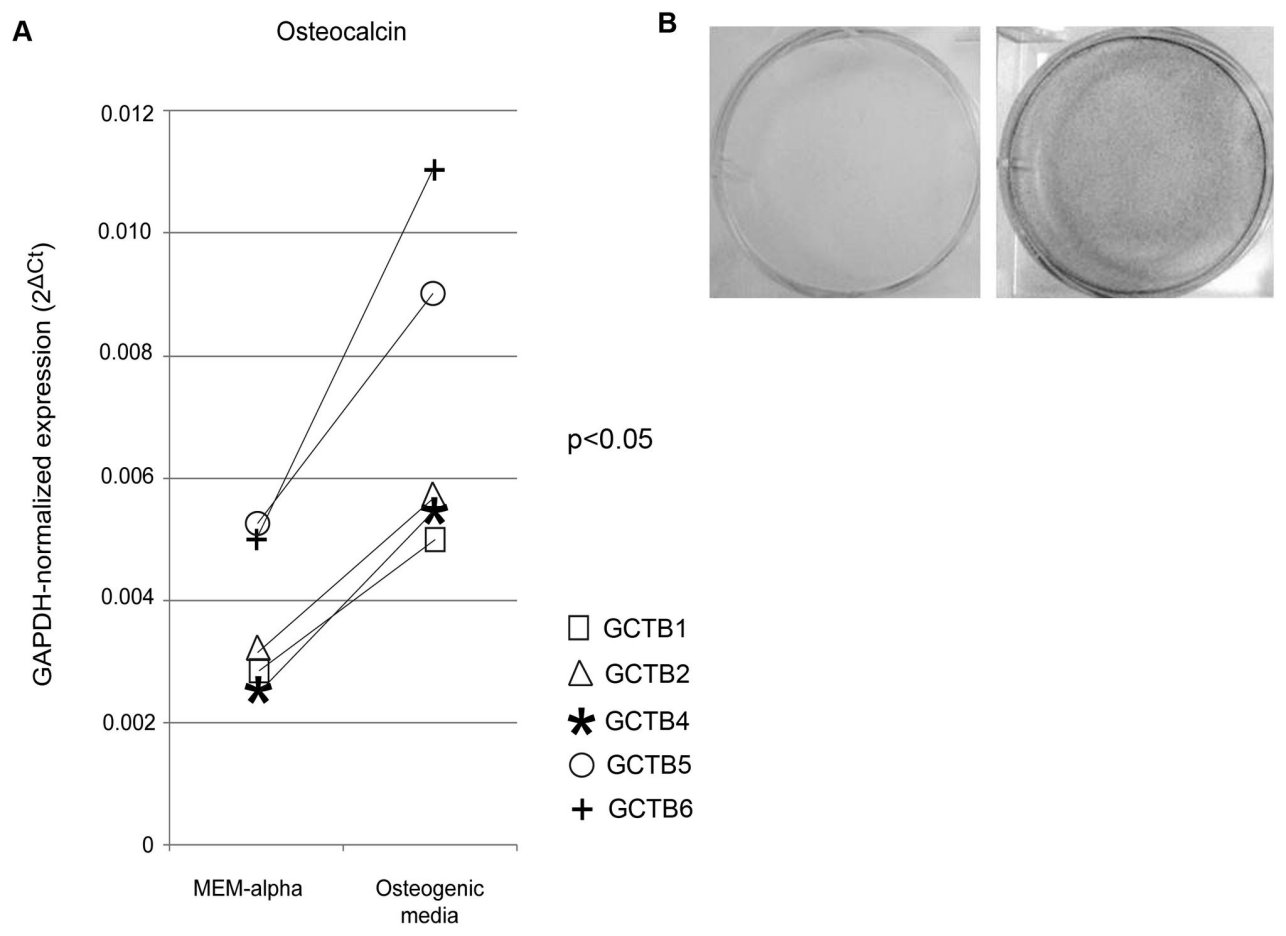
Osteogenic media increases osteoblastogenesis by GCT stromal cells. GAPDH-normalized mRNA levels of osteocalcin in cells cultured for 12 days in MEM-α or osteogenic media. (A) Osteogenic media induced osteocalcin expression (p<0.05). (B) Von Kossa staining demonstrates enhanced matrix deposition in the presence of mineralizing culture conditions.

To assess the constitutive phenotype of CD14-negative cells from the different tumors, purified RNA was prepared from freshly sorted cells and reverse transcribed to cDNA, followed by quantitative PCR to examine the expression levels of osteoblast lineage genes. Despite the use of primary freshly isolated cells, the mRNA levels for four representative osteoblast genes (Runx2, Osx, ALP, and osteocalcin) were highly variable. Several approaches were undertaken to further characterize the osteoblast lineage of the CD14-negative cells. First, sorted stromal cells were grown in the presence or absence of osteogenic media. As shown in Figure 2A, this approach induced osteocalcin expression (p<0.05), while effects on expression levels of Runx2 (p=0.43), ALP (p=0.31), and Osx (p=0.87) were variable (data not shown). Despite the variable effects of the osteogenic media on the expression of the osteoblast-associated genes, CD14-negative cells from all of the tumors tested demonstrated the capacity to form mineralized nodules as seen by positive Von Kossa staining (shown for one representative GCTB specimen in Figure 2B). These findings are consistent with the presence in this cell population of precursor cells with the capacity to differentiate into bone-forming osteoblasts.

**Figure 3:**
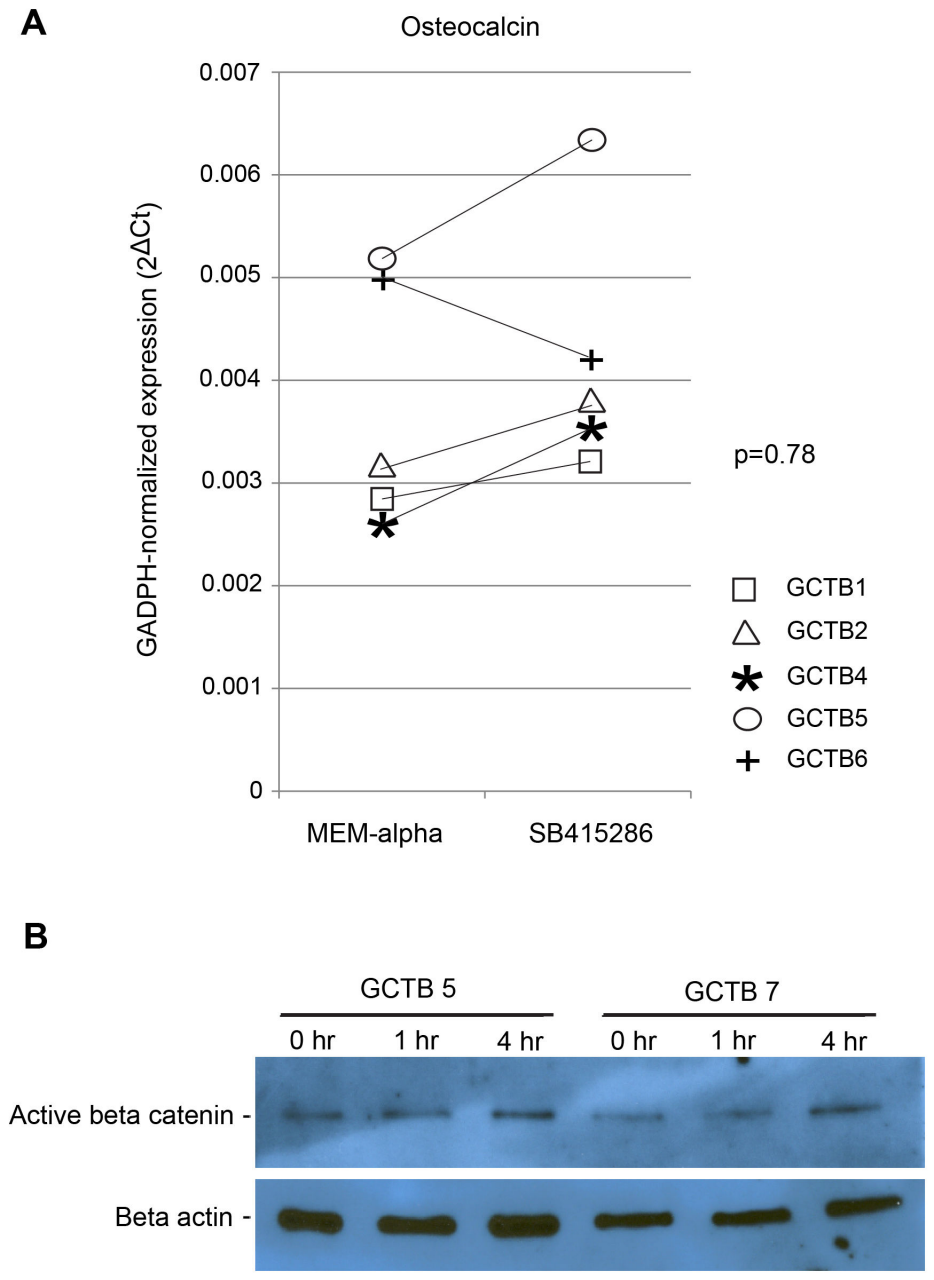
Enhanced Wnt signaling fails to alter osteoblastogenesis by GCT stromal cells. (A) GAPDH-normalized expression (qPCR) of osteocalcin mRNA levels after 12 days of growth in MEM-α with or without the Wnt signal activator, SB415286 (p= 0.78). (B) Western blot of hypophosphorylated beta-catenin after treatment of cultures with SB415286 for 0, 1, or 4 hours, indicating up-regulation of Wnt signaling.

Previous studies have shown that the differentiation of osteoblasts from mesenchymal precursors is regulated by the Wnt and BMP signaling pathways [32–36]. To determine whether activation of Wnt signaling could induce osteogenic differentiation of tumor stromal cells, we treated CD14-negative stromal cells grown in osteogenic media with the GSK3β inhibitor SB415286, which activates β-catenin and up-regulates canonical Wnt signaling [30]. As shown in Figure 3A, treatment with the Wnt pathway agonist produced variable effects on the expression levels of Runx2 (p=0.65), Osx (p=0.63) and ALP (p=0.56). This treatment increased osteocalcin levels in all tumor cell samples except GCTB 6. Western blotting of stromal cell lysates confirmed accumulation of active, hypophosphorylated β-catenin within 4 hours after SB415286 administration (Figure 3B). The magnitudes of the observed increases were small, however, and no statistical significance could be demonstrated (p=0.78).

**Figure 4:**
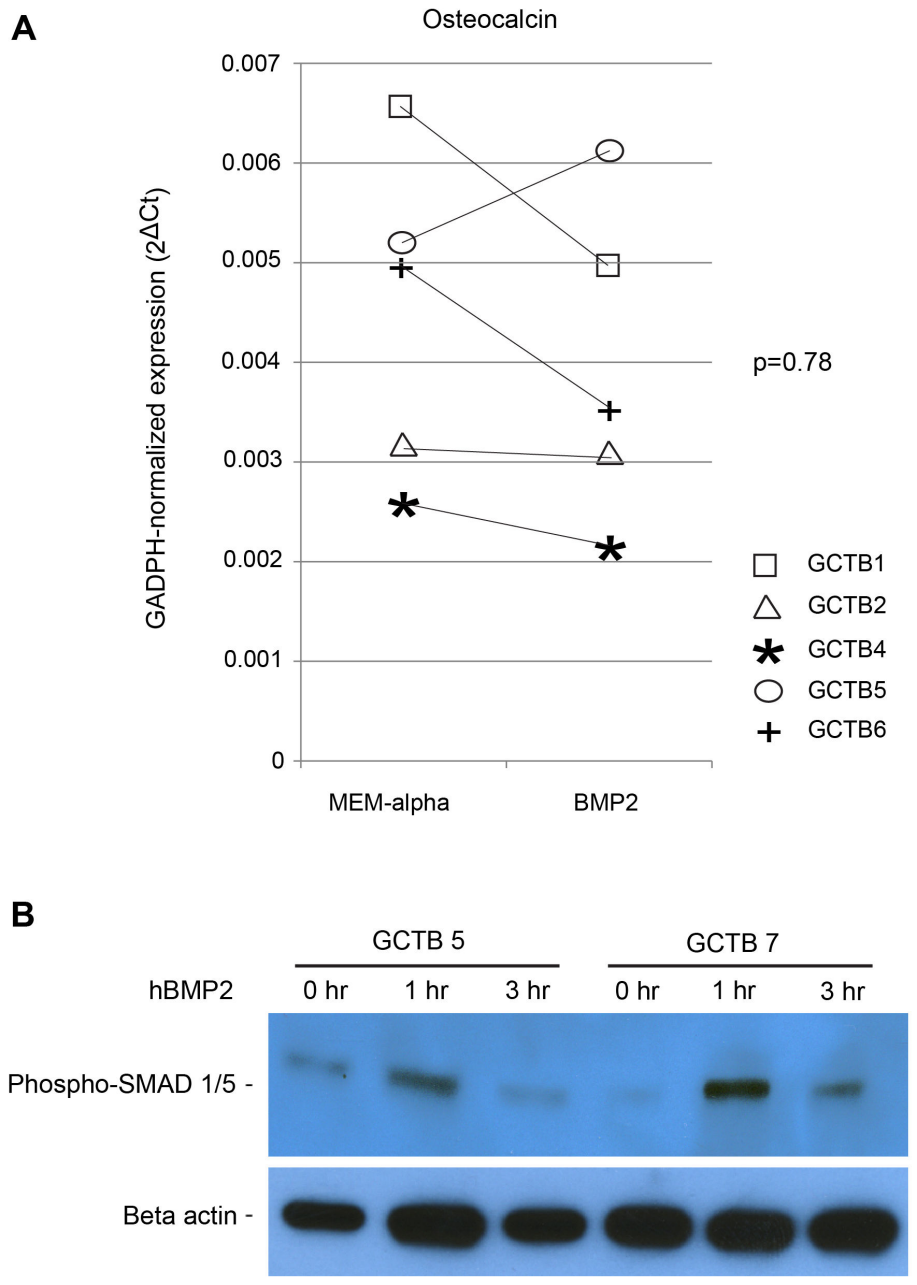
Enhanced BMP signaling fails to alter osteoblastogenesis by GCT stromal cells. (A) GAPDH-normalized expression (qPCR) of osteocalcin mRNA levels after 12 days of growth in MEM-α in the absence or presence of recombinant human BMP2. (B) Western blot analysis of phsopho-Smad 1/5 at 1 and 3 hours after treatment of cultures with vehicle or BMP2, indicating up-regulation of BMP signaling.

To further examine the osteogenic potential of the tumor stromal cells, CD14-negative stromal cells were treated with recombinant human BMP2 (rhBMP2). There were no differences in median expression levels of Osx (p=0.27), ALP (p=0.25), Runx2 (p=0.58), or osteocalcin (p=0.78), in response to BMP2 (Figure 4A), although trends towards an increase in Osx and ALP expression were noted in individual experiments (Figure 4a and data not shown). Western blotting of phosphorylated SMADs 1 and 5 confirmed active BMP signaling following exposure to rhBMP2 (Figure 4B).

### OPG and RANKL expression in GCTB stromal cells

**Figure 5:**
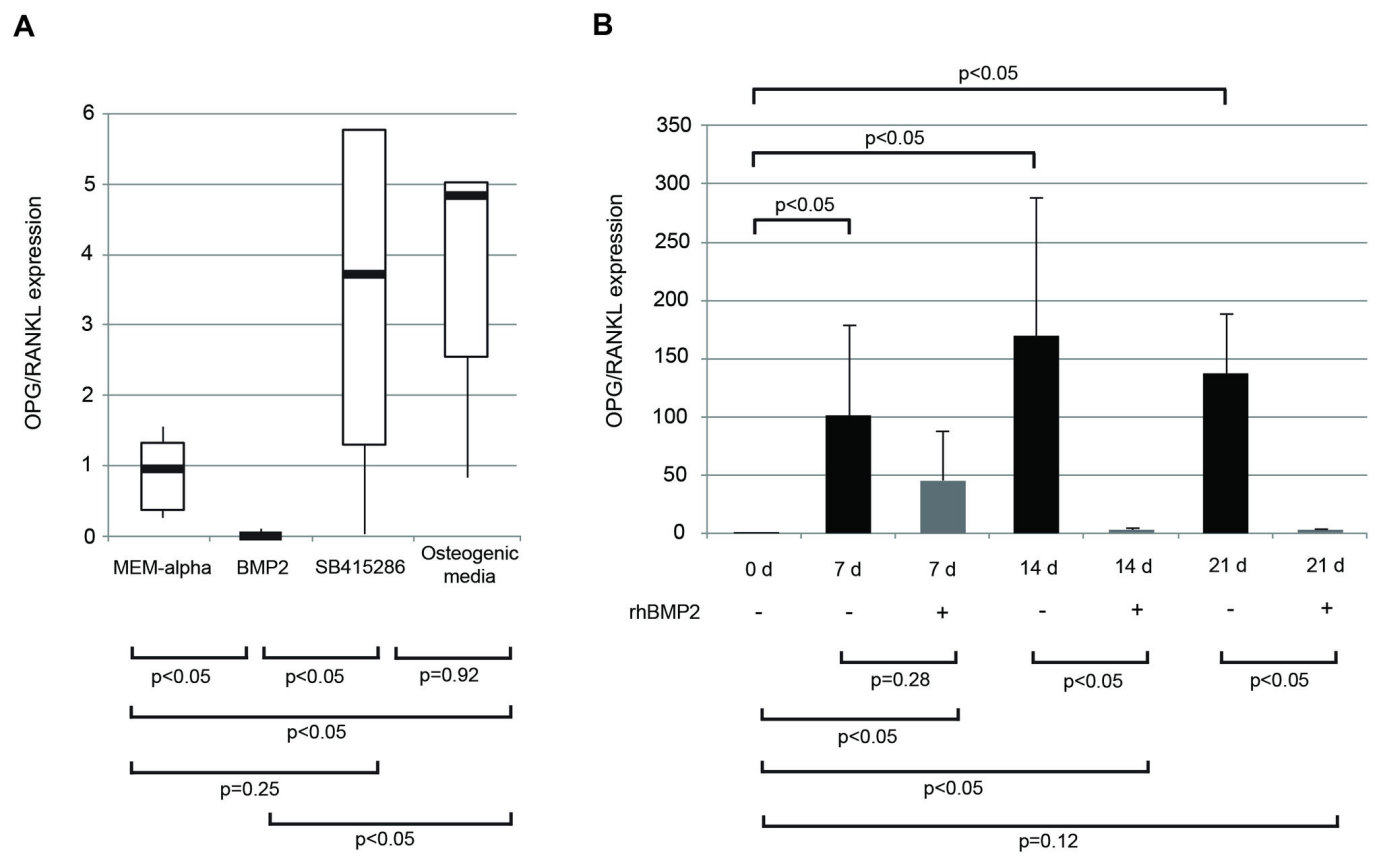
Osteoblastogenic induction selectively alters OPG and RANKL expression, while BMP2 sustains the osteoclastogenic phenotype in long-term culture. (A) Freshly isolated stromal cells were cultured for 12 days in MEM-α alone, in osteogenic media, or in medium supplemented with SB415286 or BMP2. The ratio of OPG/RANKL mRNA levels are presented relative to the ratio obtained by culture in MEM-α. While BMP2 suppresses the ratio, SB415286 increases it and osteogenic conditions fail to alter the values. The results for BMP2 treatment differs from that of cells grown in osteogenic media. (B) GCT stromal cells were cultured in the presence or absence of rhBMP-2 in MEM-α media over 21 days. Normalized OPG/RANKL ratios are presented relative to the ratio in untreated, freshly-isolated cells.

Existing evidence suggests that the pro-osteoclastogenic capacity of GCTB stromal cells is associated with a high expression ratio of RANKL/OPG [3,6,19,20,27–29]. The following studies were undertaken to characterize the pattern of RANKL and OPG expression in the freshly isolated stromal cells. We also examined the effects of osteoinduction by treatment with osteogenic media or Wnt/β-catenin and BMP pathway modulators on the expression levels and ratios of OPG and RANKL mRNA. Freshly isolated stromal cells from all tumors expressed detectible RANKL mRNA, although the levels in cells from the individual tumors varied. OPG levels were relatively low in comparison, resulting in a low OPG/RANKL ratio (Figure 5A).

As seen with culture in MEM-α, GCTB stromal cells grown in osteogenic media expressed increased levels of OPG mRNA, but the effects on RANKL mRNA levels varied (data not shown). Nevertheless, overall, the OPG/RANKL mRNA ratios were increased in comparison to control cultures grown in standard MEM-α (p<0.05) (Figure 5A). We also queried if addition of SB415286 or BMP2 would alter these same parameters. Addition of SB415286 decreased RANKL expression significantly; however, changes in OPG expression levels were highly variable in terms of magnitude and direction (data not shown). Overall, OPG/RANKL expression ratios were not significantly changed in the SB415286 treated group compared to control cultures (p=0.25), but were increased in comparison to the BMP2 group (p<0.05) (Figure 5A). Surprisingly, OPG/RANKL expression ratios were significantly decreased in the presence of BMP2 compared to MEM-α controls, SB415286, and osteogenic media treatment groups (p<0.05) (Figure 5A). Across all samples, BMP2 treatment resulted in decreased OPG mRNA levels and a trend towards increased RANKL expression levels (data not shown).

Because the above studies were terminated at the single time point of 12 days, we next sought to determine whether OPG and RANKL expression changed as a function of time, particularly following treatment with BMP2. GCTB stromal cells were freshly isolated from 3 independent samples, and grown in the presence or absence of rhBMP2 over a 21-day period (representative study shown in Figure 5B). In the absence of rhBMP2 treatment, a statistically significant increase in the OPG/RANKL expression ratio occurred within 7 days and continued to day 21 (p<0.05). Conversely, when rhBMP2 was added, the OPG/RANKL expression ratio was significantly lower than controls by 14 days, and after 21 days of BMP2 treatment, the OPG/RANKL expression ratio was unchanged compared to day 0 untreated controls.

### Effect of osteogenic media, Wnt and BMP signal activation on OPG levels in culture media

**Figure 6:**
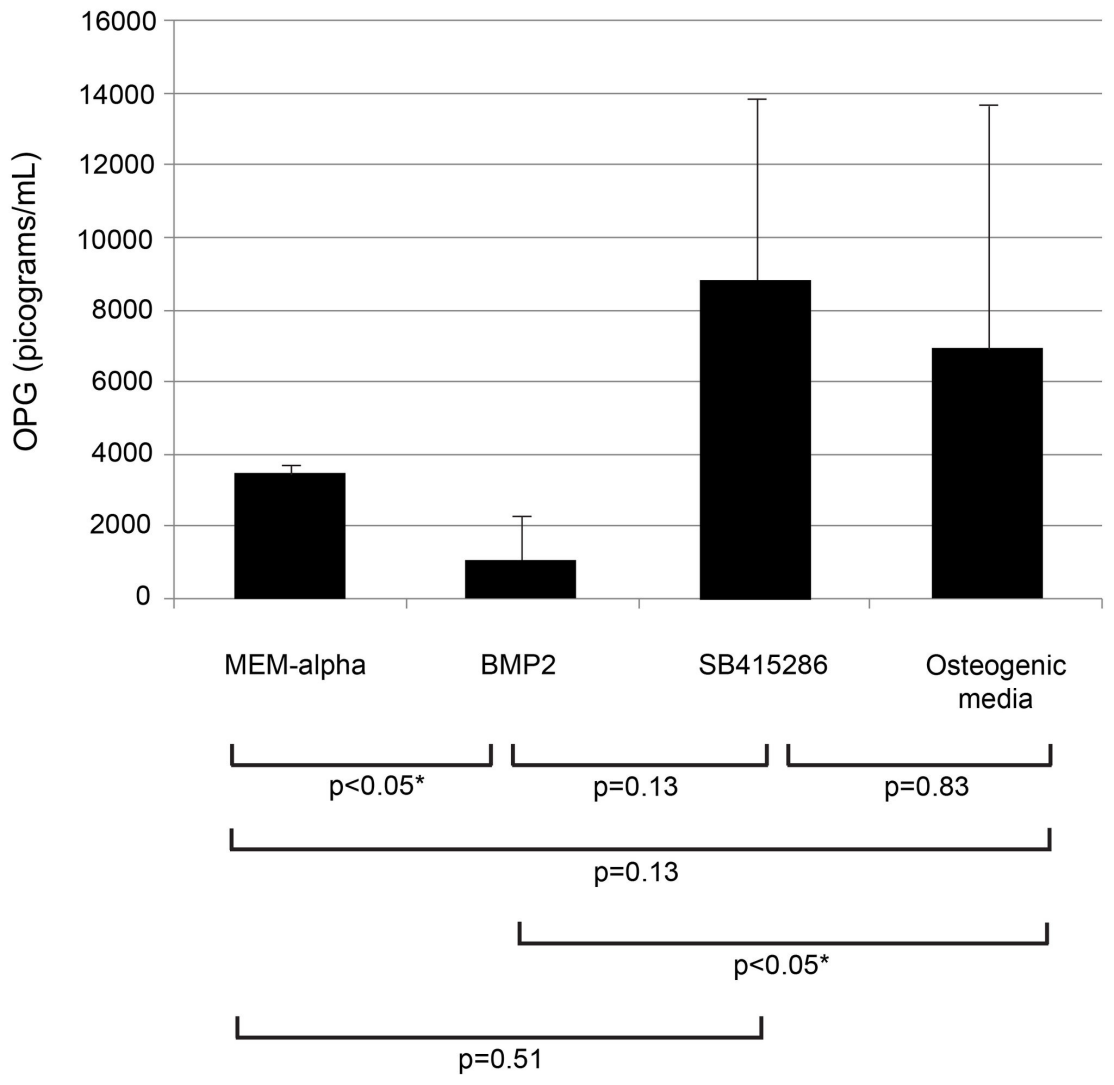
Osteoprotegerin secretion by GCT stromal cells was altered selectively by exposure to osteogenic media, BMP2, or SB415286. Stromal cells were cultured for 12 days in MEM-α, osteogenic media, or with medium supplemented with SB415286, or rhBMP2. OPG production in culture media was suppressed by BMP2 but increased in the presence of osteogenic media or SB415286 from culture days 10-12 as determined by ELISA.

To confirm that the alterations in the OPG mRNA levels described in Figure 5 were accompanied by comparable changes in OPG protein secretion, stromal cell–conditioned media were analyzed by ELISA after the various treatments. As shown in Figure 6, GCTB stromal cells grown in the presence or absence of osteogenic media demonstrated a trend towards increased production of OPG compared to controls (p=0.13). Similar to the variable effects on OPG mRNA levels of the SB415286 treatment, OPG protein levels in the culture media were highly variable and not significantly different from the other treatment groups or controls (Figure 6) (p=0.25). Addition of BMP2 resulted in a marked decrease in OPG levels compared to controls (p<0.05), SB415286 (p<0.05), and osteogenic media (p<0.05), similar to the observed effects on OPG mRNA levels. These increases were statistically significant compared to those obtained from cells cultured in osteogenic medium (p<0.05).

### Assessment of treatment conditions on osteoclast inducing activity of GCTB stromal cells

**Figure 7:**
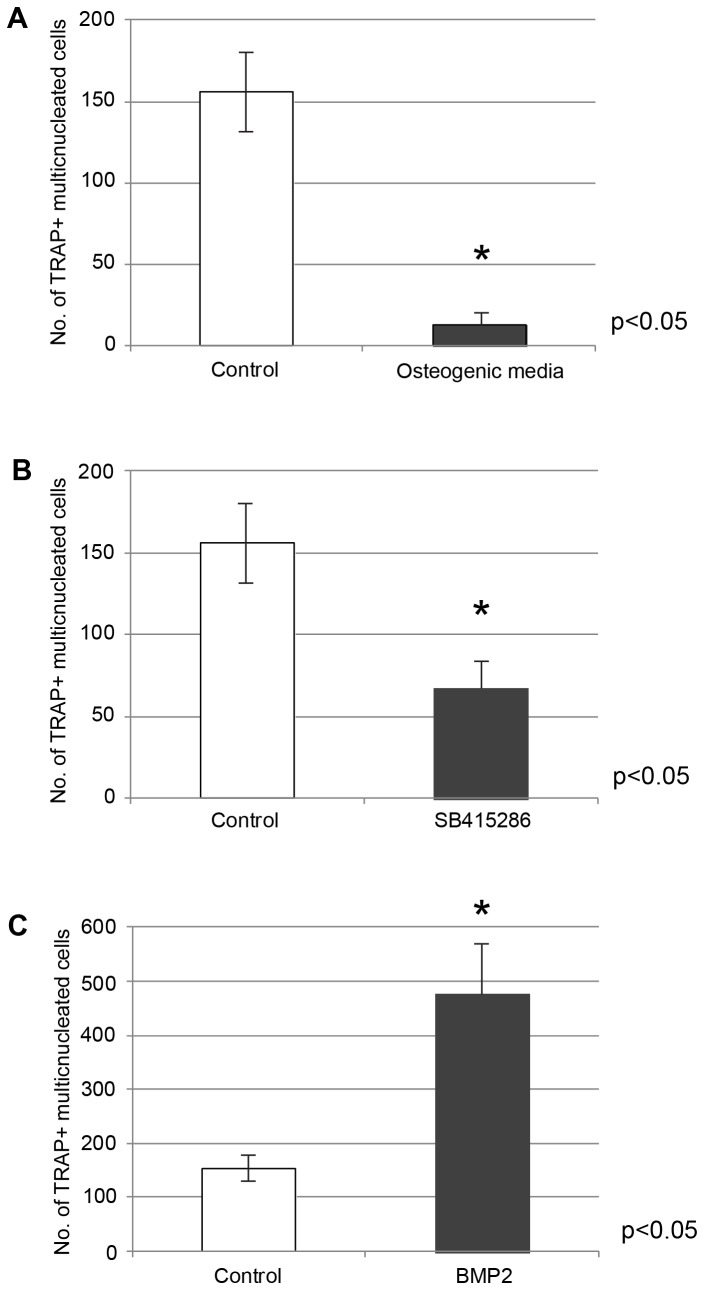
Activation of BMP2 signaling induces the osteoclastogenic potential of GCT stromal cells, while osteogenic media and SB415286 have the opposite effect. Human peripheral blood monocytes were added to GCTB stromal cells pre-exposed to 12 days of MEM-α media supplemented with (A) SB415286, (B) osteogenic media, or (C) rhBMP2. The number of osteoclasts, enumerated as TRAP+ multinucleated cells, is shown.

Co-culture of GCTB stromal cells with human peripheral blood monocytes resulted in a marked stimulation of osteoclast formation in all samples tested (data not shown), as quantified by triplicate counts of TRAP+ multinucleated giant cells. Pretreatment of the GCTB stromal cells with osteogenic media or the Wnt/β-catenin pathway activator SB415286 resulted in significant reductions in the osteoclast-inducing activity compared to the controls (p<0.05) (Figure 7A and 7B). In contrast, BMP2 pretreated GCTB stromal cells demonstrated significantly enhanced capacity to induce osteoclast formation (Figure 7C). Importantly, all changes after the various treatments correlated with their effect on the OPG/RANKL ratios.

### Demonstration of BMP receptors and putative ligands in GCTB stromal cells

**Figure 8:**
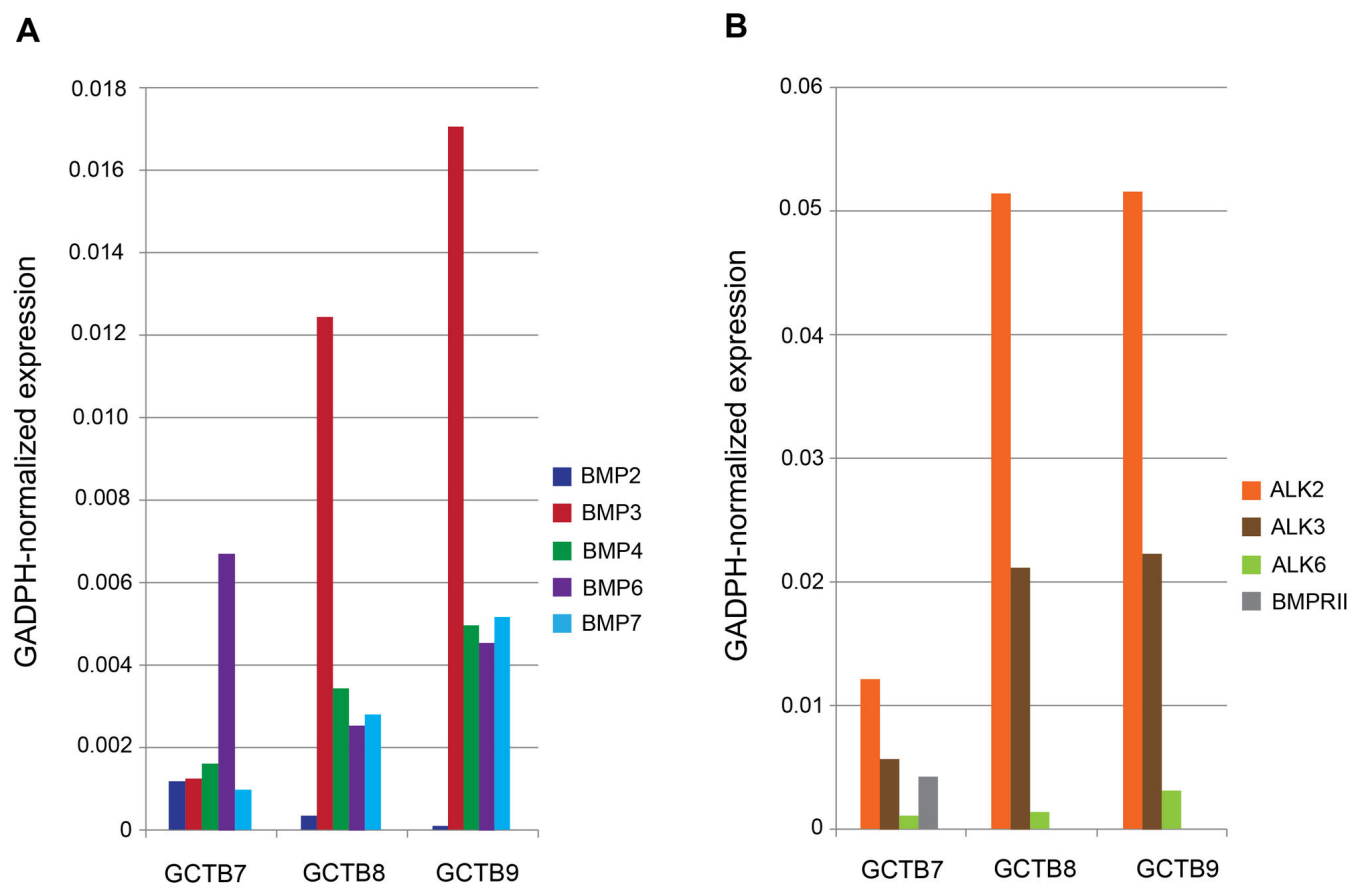
GCTB stromal cells express multiple BMPs and their ligands. Freshly isolated GCTB stromal cells from three tumors were assayed for GAPDH-normalized mRNA expression of BMPs 2, 3, 4, 6, and 7 as well as ALK2, ALK3, ALK6 and BMPRII by qPCR.

A previous publication reported the presence of mRNA for several BMPs in stromal cells cultured from GCTB [37]. Based on these observations, we performed PCR-based expression profiling of BMP pathway receptors and ligands using RNA prepared from the CD14-negative GCTB stromal cells (Figure 8). Three independent samples were analyzed. BMP2, BMP3, BMP4, BMP6, and BMP7 expression was confirmed in each of three samples tested. The putative BMP receptors, Alk2, Alk3, Alk6, were also expressed. BMPRII was present in only one of three samples. Interestingly, expression of the BMP ligands was also detected in the CD14-positive cell population from each of these tumors (data not shown).

## Discussion

In this investigation, we used histologic evaluation of GCTB and *in vitro* cell culture studies to more rigorously characterize the phenotype and lineage of the stromal cell population in GCTB, and to define the cellular and molecular mechanisms that are responsible for their unique osteoclast-inducing function within the tumor. By using freshly harvested, highly enriched GCTB cell populations that were maintained *in vitro* for a very limited period, we sought to avoid the altered phenotypic features and loss of osteoclast-inducing capacities typically exhibited by the longstanding, repeatedly passaged cultures of GCTB stromal cells used in studies to date [3–6,16,27].

It is conceivable that our strategy of extracting CD14+ monocyte-lineage cells and co-localizing MNGC’s from total dispersed cells did not effectively separate other cells known to be present in GCTB such as macrophage precursors, osteoclasts, endothelial cells, or cells of the adaptive immune system. However, GCTB stromal cells isolated using the CD14 negative selection technique (1) were morphologically consistent with cells of osteoblast-lineage; (2) were enriched in osteoblast markers; (3) were capable of undergoing terminal differentiation in the presence of mineralizing culture conditions; and (4) strongly induced osteoclastogenesis in co-culture with human monocytes. These data indicate that our method of isolation was specific for isolating a highly enriched, functional population of GCTB stromal cells. Further refinement of this isolation method is ongoing, as is an effort to validate these findings beyond the limited number of samples demonstrated herein.

Our experiments demonstrated that negatively selected, uncultured stromal cells can undergo further differentiation and matrix mineralization under pro-osteogenic conditions. Although activation of Wnt and BMP signaling pathways promoted differentiation, the baseline characteristics and responsiveness to Wnt and BMP agonists varied extensively between different tumors. The observed variability in osteoblast-associated gene expression, the effects of the various culture conditions on gene expression profiles, and the functional properties of the cells may have resulted from the heterogeneity of the patients from which specimens were obtained, as well as their varied treatment-related factors (Table 1). Furthermore, certain tumors were harvested in limited quantities such that all samples were not included in every experiment. This heterogeneity, evident in the histologic features of the harvested tumor tissue and in the *in vitro* studies of the CD14+ cell fractions, may have also contributed to the variable functional properties of the stromal cell isolates. Importantly, our data provide further insights into the mechanisms by which the stromal cells play a pivotal role in the recruitment and induction of myeloid lineage cells to form the characteristic osteoclast-like giant cells of the tumors [6–10].

Previous assertions that GCTB stromal cells are of osteoblast lineage were based on their expression of osteoblast and pre-osteoblast molecular markers [16,20,22–26]. The expression of multiple osteoblast- and pre-osteoblast-associated genes and gene products in our isolated, unpassaged stromal cells provide additional support for this conclusion. Further, we demonstrated that bone formation could be induced in stromal cells by culture in osteogenic medium. Of note, this characteristic was displayed by the stromal cell populations isolated from all of the harvested tumors, supporting the general concept that cells of osteoblast lineage do, in fact, exist within the tumor stromal cell population. The absence of mineralized tissue in the original tumor and the relatively low levels of expression of osteocalcin, a marker of late-stage osteoblast differentiation, are consistent with an arrest of the cells in an intermediate stage of differentiation with failure to reach terminal stages of osteoblast differentiation. Whether this is related to an intrinsic property of the cells or the unique tumor microenvironment and interaction with other cellular components of the tumor remains unclear. Interestingly, other studies have shown that osteoclasts are a source of products that can either inhibit or enhance osteoblast differentiation [38–42].

Previous studies have provided evidence that GCTB stromal cells exhibit osteoclastogenic activity when co-cultured with monocytic osteoclast precursors [23,43–47]. Such activity has been attributed to stromal cell production of numerous factors involved in recruitment and induction of osteoclast differentiation and activation, including RANKL [3,6,19,20,27–29]. Other potent osteoclastogenic proteins have been reported in GCTB, including M-CSF [6] and its structural homolog interleukin 34 [48], as well as Siglecs [49]. These latter membrane-bound glycoproteins co-activate the immunoreceptor tyrosine-based activation motif (ITAM) signaling pathway responsible for intracellular calcium signaling, a key component of induction of nuclear factor of activated T cells, cytoplasmic 1 (NFATc1), the master transcriptional regulator of the osteoclast [49]. Our freshly harvested and purified stromal cell populations exhibited potent osteoclast-inducing activity when co-cultured with peripheral blood mononuclear cells or with the CD14+ myeloid lineage cells from the original tumors. Consistent with previously published data, a rapid loss of osteoclastogenic capacity occurred in our cell populations with prolonged time in culture or repeated passaging [24,27,47]. We speculate that the decrease in RANKL production and up-regulation of OPG provide a plausible explanation for the loss of osteoclast-inducing activity.

Culture of our CD14-negative stromal cells under osteoblast-inducing conditions resulted in a striking reversal in the OPG/RANKL ratios involving both up-regulation of OPG and downregulation of RANKL. Both the osteogenic media and the activation of the Wnt/β-catenin signal pathway by the GSK-3 inhibitor enhanced OPG expression and increased the OPG/RANKL ratio compared to control. Although the mechanism by which the osteogenic media produced these effects is not known, we speculate that the effects of SB415286 were related to increased expression of dephosphorylated β-catenin (Figure 4). Others have shown that β-catenin regulates OPG gene expression [50–53] by interaction with the transcription factors T-cell factor/Lymphoid Enhancer Factor (TCF/LEF) [33]. These transcription factors also regulate the expression of osteoblast-associated genes, including Runx2, Osterix [34], and osteocalcin [35].

The loss of osteoclast-inducing capacity after induction of differentiation of the stromal cells into a more definitive osteoblast phenotype has important and potentially promising clinical implications for the therapeutic inhibition of GCTB osteolytic activity. In a recent study of patients with refractory, non-resectable GCTB, treatment with denosumab, a human monoclonal antibody that binds and inhibits RANKL, resulted in depletion of tumoral giant cells, accompanied by inhibition of osteolytic progression in a majority of patients [19]. In several patients, induction of local regions of bone formation within the tumors was evident, which may have been related to tumor stromal cell differentiation into osteoblastic cells. These findings were recently validated by others [18]. Although the lytic, destructive behavior of GCTB is attributable to robust osteoclastogenesis from monocyte precursors, sole targeting of the myeloid components of the tumor may be insufficient to suppress stromal cell-mediated disease progression in the long term. Thus, stromal cell-directed therapeutic strategies remain a highly relevant approach for treatment of GCTB.

In contrast to the findings with osteogenic media and Wnt/β-catenin pathway activation, we found that BMP treatment of stromal cells inhibited the effects of long-term culture on the increase in the OPG/RANKL ratio. Importantly, these results were accompanied by maintenance of the osteoclast–inducing capacity of the stromal cells even after prolonged culture. BMP ligands are members of the Transforming Growth Factor Beta superfamily of proteins and initially bind to the Type II receptor resulting in recruitment, dimerization and phosphorylation of the Type I receptor. Receptor dimerization drives formation of R-SMAD/co-SMAD complexes that, upon nuclear translocation, function as transcription factors regulating cell proliferation, apoptosis, and differentiation [36,54]. The effects of BMP signal activation on OPG and RANKL expression in mesenchymal-lineage have yet to be fully elucidated [55–57]. The suppression of OPG and simultaneous up-regulation of RANKL expression in response to BMP2 was seen in C2C12 cells, which are regarded as pluripotent mesenchymal precursors, and has been suggested as a potential mechanism by which early osteoblast lineage cells stimulate osteoclastogenesis [58]. Interestingly, BMP/SMAD-pathway activation has been associated with osteolysis in sheep and non-human primate models of spinal fusion and fracture repair [55,58]. In these models, osteolysis was noted within days after introducing hyperphysiologic levels of BMP2 into the intramedullary canal, with subsequent robust bone formation up to 8 weeks later. Although we used a significantly lower BMP2 concentration, these *in vivo* studies support the concept that BMP/SMAD signal activation can induce osteolysis.

Recent data have suggested that activation of the BMP/SMAD signal pathway also may enhance osteoclastogenesis through direct effects on osteoclast precursors [59,60]. In our studies, osteoclast precursors were not exposed to exogenous BMP2, so as to avoid potential direct effects of BMP2. Since BMP signaling can keep stromal cells in their tumoral pro-osteoclastogenic state of arrested differentiation, it is possible that aberrant BMP signaling may represent a key component of GCTB pathophysiology. Our findings were consistent with this possibility, as BMP ligands and receptors were expressed in both stromal and myeloid cell populations, suggesting that BMP pathway modulation may represent an additional potential differentiation therapy for GCTB. Future clinical application of this therapeutic strategy raises other issues. Accurate diagnosis is essential, as cancers that are difficult to distinguish from GCTB, such as giant-cell rich osteosarcoma, may be stimulated and worsen prognosis [61,62]. Finally, such treatment does not exclude anti-resorptive therapy.

In summary, our studies confirm that GCTB stromal cells are of osteoblast lineage, as evidenced by their capacity for expression of definitive markers of differentiated osteoblasts and initiation of bone mineralization under osteogenic conditions. We speculate that the arrest of the stromal cells in a state of early osteoblast differentiation is associated with up-regulation of RANKL production and that the production of this potent osteoclast-inducing factor accounts for the unique capacity of the tumors to form multinucleated osteoclasts and to induce osteolysis. Our findings that activation of the Wnt/β-catenin and BMP pathways differentially modulate the phenotype and osteoclast-inducing activity of the stromal cells has potential clinical applications for treating patients with refractory destructive GCTB.
